# Gilteritinib Suppresses Hepatocellular Carcinoma Cells in Association with Reduced Calpain-1 Expression and Inhibition of STAT3/AKT Signaling

**DOI:** 10.3390/biomedicines14102137

**Published:** 2026-09-22

**Authors:** Sijing Chen, Yunyang Zhao, Yunqi Pan, Zhihui Zhao, Zhonghua Ma, Yujie Wei, Ting Liu, Limei Shuai, Wenjiao Gu, Shuangyi Li, Junjie Shao, Ying Jiang, Ailifeire Maimaitituerxun, Xinghuai Wang, Xiaochun Zhou, Futian Tang

**Affiliations:** 1 School of Basic Medical Sciences, Lanzhou University, Lanzhou 730000, China; 2 Gansu Province Key Laboratory of Environment Oncology, Lanzhou University Second Hospital, Lanzhou 730030, China; 3 Department of Cardiovascular Disease, The Second Hospital & Clinical Medical School, Lanzhou University, Lanzhou 730000, China; 4 Department of Urology, Minqin County People’s Hospital, Minqin 733300, China; 5 Department of Nephrology, Lanzhou University Second Hospital, Lanzhou 730030, China

**Keywords:** gilteritinib, hepatocellular carcinoma, calpain-1, STAT3/AKT signaling pathway, apoptosis

## Abstract

**Background/Objectives**: Hepatocellular carcinoma (HCC) is a leading cause of cancer-associated mortality worldwide, and current targeted therapeutic options remain inadequate. Gilteritinib, a clinically approved tyrosine kinase inhibitor, has shown antitumor activity in hematologic malignancies; however, its therapeutic role and underlying mechanisms in HCC remain unclear. **Methods**: The effects of gilteritinib on HCC were evaluated in Hepa1-6 and Huh-7 cells. Cell proliferation, migration, invasion, apoptosis, and cell cycle distribution were assessed by CCK-8, wound healing, Transwell, and flow cytometry assays. Protein expression and total calpain activity were assessed by Western blotting and enzymatic activity assays. Surface plasmon resonance (SPR) analysis was used to examine the cellular association of gilteritinib with HCC cells. Antitumor efficacy and safety were further evaluated in a Hepa1-6 allograft mouse model. **Results**: Gilteritinib significantly inhibited the proliferation, migration, and invasion of HCC cells and induced G2/M-phase arrest and apoptosis. Gilteritinib treatment was associated with reduced calpain-1 expression and total calpain activity. These changes were accompanied by decreased phosphorylation of STAT3 and AKT, downregulation of Bcl-2, and upregulation of Bax. In vivo, gilteritinib markedly suppressed tumor growth in Hepa1-6 tumor-bearing mice. **Conclusions**: Collectively, gilteritinib exerts antitumor effects against HCC both in vitro and in vivo, and these effects are closely associated with reduced calpain-1 expression and inhibition of STAT3/AKT signaling. These findings suggest that gilteritinib has therapeutic potential in HCC and highlight calpain-1 as a candidate downstream correlate worthy of further mechanistic study.

## 1. Introduction

Hepatocellular carcinoma (HCC) accounts for 75–85% of all primary liver cancers and is also a leading contributor to cancer-associated deaths worldwide [1,2]. It represents a serious threat to human health, particularly in Asia, where over 70% of new cases occur [3]. HCC lacks specific symptoms and biomarkers in its early stages; consequently, many patients are diagnosed at intermediate or advanced stages [4,5] and miss the optimal window for curative treatment. HCC is prone to intrahepatic dissemination and extrahepatic metastasis, features closely associated with epithelial–mesenchymal transition (EMT), neovascularization, and dynamic changes in the tumor microenvironment [6]. Despite recent advances in surgery, liver transplantation, and targeted treatment [7], the prognosis for HCC patients remains poor, largely due to frequent tumor recurrence, metastasis, and drug resistance [8]. Currently, molecular targeted therapy serves as a cornerstone for managing advanced HCC [9]. Multi-targeted TKIs, such as sorafenib and lenvatinib, can inhibit both cancer cell proliferation and angiogenesis [10,11]. Nonetheless, the clinical benefits of currently approved agents remain limited. The vast majority of patients eventually develop acquired drug resistance and experience drug-related adverse reactions [12,13]. There is an urgent need to identify new therapeutic targets and develop effective targeted agents in HCC.

Calpain-1, a calcium-dependent cysteine protease, has been increasingly recognized as an important regulator of tumor progression [14]. Calpain-1 participates in multiple cellular processes, including cell proliferation, migration, apoptosis, and cytoskeletal remodeling [15]. Accumulating evidence suggests that calpain-1 is aberrantly activated in various cancers, including HCC, where it contributes to tumor growth, metastasis, and resistance to cell death [16,17,18]. Mechanistically, calpain-1 can modulate STAT and AKT signaling pathways, which are crucial drivers of malignant cell survival [15,19]. Dysregulation of STAT and AKT signaling is frequently observed in HCC and is closely associated with tumor progression and poor clinical prognosis [20,21,22,23,24,25]. By modulating cellular signaling, including the PI3K/AKT/mTOR and JAK/STAT pathways, the calpain system governs cell proliferation, apoptosis, and cell migration [26]. Therefore, targeting calpain-1 may be a promising therapeutic strategy for HCC, potentially disrupting these critical oncogenic pathways and improving patient outcomes.

Gilteritinib, an oral TKI, was initially approved for relapsed or refractory acute myeloid leukemia (AML) harboring FLT3 internal tandem duplications or tyrosine kinase domain point mutations [27]. In AML, gilteritinib inhibits mutant FLT3 autophosphorylation, suppressing downstream signaling (STAT5, MAPK/ERK, PI3K/AKT) and inducing apoptosis [28]. However, emerging evidence suggests a broader antitumor spectrum for gilteritinib beyond hematologic malignancies. Recent studies have reported its antitumor activity in solid tumors, including esophageal, ovarian, and gastric cancers. Mechanistically, gilteritinib modulates multiple oncogenic processes, including apoptosis, cell cycle progression, and various signaling cascades [29]. Together, these studies indicate that gilteritinib has broad-spectrum antitumor activity. Nevertheless, its therapeutic potential and underlying mechanisms in HCC remain largely unclear. In particular, whether gilteritinib affects calpain-1 and its downstream signaling pathways has not been investigated.

In the present study, we explored the antitumor effects and molecular mechanisms of gilteritinib in HCC. Using Hepa1-6 and Huh-7 cells, we evaluated the effects of gilteritinib on cell proliferation, migration, invasion, cell cycle distribution, and apoptosis. We further investigated whether gilteritinib treatment affects calpain-1 expression and total calpain activity and assessed its effects on STAT3 and AKT signaling pathways. Whole-cell SPR analysis was performed to assess the cellular association of gilteritinib with HCC cells. In addition, the antitumor efficacy and safety of gilteritinib were evaluated in a Hepa1-6 allograft mouse model.

Our findings suggest that gilteritinib exerts antitumor effects against HCC and that these effects are associated with reduced calpain-1 expression and total calpain activity, inhibition of STAT3/AKT signaling, increased apoptosis, and suppressed tumor growth. Although the causal role of calpain-1 remains to be established, these results suggest that calpain-1 may serve as a candidate biomarker or potential downstream correlate of gilteritinib’s effects. These findings also support the further evaluation of gilteritinib as a therapeutic option for HCC.

## 2. Materials and Methods

### 2.1. Cell Lines and Cell Culture

Hepa1-6 and Huh-7 cells were purchased from Zeesan Biotechnology Co., Ltd., Wuhan, China. For STR identification, genomic DNA was extracted from Hepa1-6 and Huh-7 cells (>1 × 10^6^ cells) using the Tsingke Animal Genomic DNA Extraction Kit (TSP210-200, Tsingke Biotechnology Co., Ltd., Beijing, China), and the STR (short tandem repeat) and Amelogenin loci were amplified using the New Seabio NuHi SU9 dNTP Mix (NH9347, New Seabio Biotechnology Co., Ltd., Suzhou, China). The resulting profiles were analyzed via the Cellosaurus CLASTR tool and cross-referenced with databases from ATCC, DSMZ, JCRB, RIKEN, and published data. The analyses confirmed a complete match between the submitted cell lines and the authentic Hepa1-6 and Huh-7 reference cell lines. Cells were cultured in DMEM (Gibco, Grand Island, NY, USA) supplemented with 10% FBS (Gibco, Grand Island, NY, USA) and 1% penicillin–streptomycin at 37 °C in a 5% CO_2_ atmosphere. For all experiments, cells were collected in the logarithmic growth phase to ensure optimal viability and consistent assay performance.

### 2.2. Drug Preparation and Treatment

Gilteritinib was purchased from TargetMol Chemicals Inc. (Boston, MA, USA). A 10 mM stock of gilteritinib in DMSO (dimethyl sulfoxide) was prepared and stored at −20 °C. Working dilutions were prepared on the day of use by adding culture medium to the stock solution. Throughout the study, cells were treated with gilteritinib at the concentrations indicated for each assay, while control cells received an equal volume of DMSO. The final concentration of DMSO in all experimental groups was kept below 0.1% to avoid solvent-related cytotoxic effects.

### 2.3. Cell Viability Assay

Cell viability was measured using a Cell Counting Kit-8 (G4103-1ML, Servicebio, Wuhan, China) according to the manufacturer’s instructions. Briefly, Hepa1-6 and Huh-7 cells were seeded into 96-well plates at 5000 cells per well and incubated overnight. Thereafter, cells were treated with serial dilutions of gilteritinib (0–100 μM) for 24 h. After adding 10 μL CCK-8 to each well and incubating at 37 °C for 30 min, absorbance was measured at 450 nm using a microplate reader (Bio-Rad Laboratories Inc., Hercules, CA, USA). Relative cell viability and IC_50_ values were calculated using GraphPad Prism by normalizing to the control group readings.

### 2.4. Wound Healing Assay

Cell motility was examined by wound healing assays. Approximately 90% confluent monolayers of Hepa1-6 and Huh-7 cells in 6-well plates were scratched in a straight line with a sterile pipette tip. After floating cells were removed by washing with PBS, fresh serum-free medium containing gilteritinib or vehicle control was added. Serum-free medium was used to suppress cell proliferation, ensuring that wound closure reflected cell migration. Micrographs of the scratched fields were taken at 0, 24, and 48 h under an inverted microscope. Wound closure was quantified with Fiji (ImageJ, Version 1.54p, National Institutes of Health, Bethesda, MD, USA) as (A_0_ − A_24/48_)/A_0_ × 100%, where A_0_ is the area at 0 h and A_24/48_ is the area at 24 or 48 h.

### 2.5. Transwell Migration and Invasion Assays

Transwell chambers (Corning, NY, USA, 8.0-μm pore size) were used for more detailed analysis of migration and invasion. For migration assays, cells (5 × 10^4^ in 200 μL serum-free medium) were seeded into the upper chamber. The lower chamber was filled with medium supplemented with 10% FBS as a chemoattractant. For invasion assays, the upper surface of the membrane was coated with Matrigel (Corning; diluted 1:8 in serum-free medium). All Matrigel handling was performed on ice. Matrigel solution (60 μL per insert) was added to the upper chamber and allowed to polymerize at 37 °C for 60 min. Excess Matrigel was then gently aspirated, and 100 μL of serum-free medium was added to each insert, followed by incubation at 37 °C for 30 min to equilibrate the membrane. After the medium was removed, HCC cells (5 × 10^4^ in 200 μL of serum-free medium) were seeded into the upper chamber. After 24 h, cells remaining on the upper surface of the membrane were gently removed with a cotton swab. Cells that had migrated or invaded through the membrane to the lower surface were fixed with paraformaldehyde, stained with 0.1% crystal violet for 15 min, photographed under a microscope, and counted using Fiji (ImageJ software, Version 1.54p, National Institutes of Health, Bethesda, MD, USA).

### 2.6. Apoptosis Analysis

For apoptosis analysis, an Annexin V-FITC/propidium iodide (PI) detection kit (Liankebio, Hangzhou, China) was employed. Hepa1-6 and Huh-7 cells were seeded in 6-well plates and treated with gilteritinib (0, 5, or 10 μM) or vehicle control for 24 h. Both floating and adherent cells were harvested, washed twice with cold PBS, and resuspended in binding buffer. Cells were then stained with Annexin V-FITC and PI according to the manufacturer’s protocol and incubated for 15 min at room temperature in the dark. Apoptotic cells were analyzed by flow cytometry, with at least 1 × 10^4^ events acquired per sample. Early (Annexin V^+^/PI^−^) and late (Annexin V^+^/PI^+^) apoptotic cells were quantified, and the total percentage of apoptotic cells was calculated using FlowJo software (Version 10.8.1, Tree Star, Ashland, OR, USA).

### 2.7. Cell Cycle Analysis

Cell cycle distribution was examined by flow cytometry using a cell cycle staining kit (Liankebio, Hangzhou, China). Cells were seeded in 6-well plates and treated with gilteritinib (0, 5, or 10 μM) or vehicle control for 24 h. After treatment, cells were harvested, washed twice with cold PBS, and fixed in 70% ethanol at 4 °C overnight. The fixed cells were then washed and incubated with PI/RNase staining solution for 30 min at room temperature in the dark. DNA content was analyzed by flow cytometry, and at least 1 × 10^4^ events per sample were recorded. Doublets were excluded by gating on FL2-A versus FL2-W, and the percentages of cells in the G0/G1, S, and G2/M phases were determined using FlowJo software (Version 10.8.1, Tree Star, Ashland, OR, USA).

### 2.8. Western Blot Analysis

HCC cells in the logarithmic growth phase were treated with various concentrations of gilteritinib or vehicle control for 24 h. After treatment, the medium was removed, and cells were washed twice with PBS prior to protein extraction. Then, HCC cells were lysed in RIPA lysis buffer (P0013B, Beyotime, Shanghai, China) supplemented with 1% protease inhibitor cocktail (ST506-2, Beyotime, Shanghai, China) and 1% phosphatase inhibitor cocktail (P1081, Beyotime, Shanghai, China) on ice for 30 min. For tumor tissues, approximately 30 mg of frozen tissue was homogenized in 300 µL of RIPA lysis buffer supplemented with protease and phosphatase inhibitors using a tissue grinder. The homogenate was then incubated on ice for 30 min. Lysates were centrifuged at 12,000× *g* for 15 min at 4 °C, and supernatants were collected. Protein concentration was determined using a BCA assay kit (PC0020, Solarbio, Beijing, China). The protein concentrations and volume were normalized. The samples were then mixed with 5× SDS-PAGE loading buffer (P1040, Solarbio, Beijing, China) and denatured at 70 °C for 10 min in a thermo-shaker prior to SDS-PAGE separation.

Equivalent amounts of total protein (10 μg per lane) were separated by SDS-PAGE using 10% separating gels and 5% stacking gels and transferred onto PVDF membranes (IPVH00010, Millipore, 0.45 μm, Darmstadt, Germany). Membranes were blocked with 5% non-fat milk in TBST (Tris-buffered saline containing 0.1% Tween-20) for 1 h at room temperature, followed by overnight incubation at 4 °C with primary antibodies. For phospho-specific antibodies (p-STAT3 and p-AKT), 5% BSA in TBST was used for blocking and antibody dilution. Primary antibodies used were as follows: calpain-1 (1:5000; ab28258, Abcam), STAT3 (1:1000; GB11176, Servicebio), p-STAT3 (Tyr705; 1:1000; GB150001, Servicebio), AKT (1:1000; GB15689, Servicebio), p-AKT (Ser473; 1:1000; GB150002, Servicebio), Bcl-2 (1:1000; AF6139, Affinity Biosciences), Bax (1:1000; AB026, Beyotime Biotechnology), GAPDH (1:10,000; GB15004, Servicebio), and β-actin (1:5000; GB15003, Servicebio). After three washes with TBST, membranes were incubated with HRP-conjugated secondary antibodies (goat anti-rabbit IgG, 1:5000, ZB-5301, ZSGB-BIO; goat anti-mouse IgG, 1:5000, A1089, Beyotime) for 1 h at room temperature. Immunoreactive signals were visualized using ECL substrate (BL523B, Biosharp) and a chemiluminescence imaging system. Band intensities were quantified using ImageJ software (NIH, Bethesda, MA, USA), and target protein expression was normalized to the corresponding loading control (GAPDH or β-actin). All Western blot experiments were repeated at least three times independently; representative blots are shown in the figures.

### 2.9. Calpain Activity Assay

Calpain activity in cell and tumor lysates was assessed using a commercial fluorogenic kit (QIA120, Sigma-Aldrich, St. Louis, MO, USA), according to the manufacturer’s instructions. HCC cells were treated with gilteritinib for 24 h, then harvested by trypsinization and centrifuged at 1000 rpm for 5 min. The cell pellets were washed twice with ice-cold PBS and lysed in the provided lysis buffer on ice for 30 min. After centrifugation at 12,000 rpm for 15 min at 4 °C, the supernatants were collected as cellular protein extracts. For tumor tissues, approximately 50–100 mg of frozen tumor tissue was minced on ice, homogenized in 500–1000 μL of lysis buffer using a tissue grinder, incubated on ice for 30 min with intermittent vortexing, and centrifuged at 12,000 rpm for 15 min at 4 °C. The supernatants were collected as tumor protein extracts. Protein concentrations of all lysates were determined using a BCA assay. Then, 50 μL of each protein supernatant was incubated with 100 μL of Activation Buffer (containing Ca^2+^ and TCEP; QIA120, Sigma-Aldrich) or 100 μL of Inhibition Buffer (containing the calcium chelator BAPTA; QIA120, Sigma-Aldrich) along with the fluorogenic substrate Suc-LLVY-AMC for 15 min at room temperature. Fluorescence was measured using a microplate reader with excitation at 380 nm and emission at 460 nm. Net calpain-specific activity was calculated by subtracting the background fluorescence (Inhibition Buffer) from the total fluorescence (Activation Buffer). Results were expressed as fold change relative to the control group. A purified human calpain-1 standard (QIA120, Sigma-Aldrich) was included as a positive control in each experiment to ensure assay reliability. It should be noted that this assay measures total calpain activity (calpain-1 and calpain-2) rather than calpain-1 exclusively. However, the parallel changes in calpain-1 protein expression and total calpain activity observed in our study suggest that calpain-1 substantially contributes to the measured activity.

### 2.10. SPR Analysis

SPR analysis was performed to evaluate the association of gilteritinib with HCC cells. The SPR experiments were performed using a BI-SPR instrument (Biosensing Instrument Inc., Tempe, AZ, USA). Hepa1-6 or Huh-7 cells were cultured on SPRM sensor chips (gold-coated glass slides) until they reached 30–70% confluence and were then fixed with 4% paraformaldehyde for 15 min. Prior to each experiment, the sensor chip was warmed to room temperature and mounted onto the prism stage with refractive index matching oil, and the flow cell was assembled. The flow system was purged with filtered, degassed running buffer at a flow rate of 80 μL/min, and the cells were allowed to stabilize under continuous buffer flow for 30 min. The SPR angle was then set, and regions of interest (ROIs) covering both cell-covered and cell-free (bare) areas of the chip were defined to enable reference subtraction. A calibration injection of 95% running buffer was applied to normalize the SPR response across the ROIs. Gilteritinib was serially diluted in running buffer to final concentrations of 1, 10, 100, 1000, and 10,000 nM, with the DMSO concentration in the samples matched to that of the running buffer (<0.1%). The samples were injected sequentially from the lowest to the highest concentration (kinetic titration), with an association (exposure) time of 180 s and a dissociation (delay) time of 180 s. Blank injections of running buffer were included for blank subtraction. SPR sensorgrams were recorded in real time using ImageSPR software (Version 2.4, Biosensing Instrument Inc., Tempe, AZ, USA). Kinetic parameters, including the association rate constant (*k*_a_), the dissociation rate constant (*k*_d_), and the apparent equilibrium dissociation constant (*K*_D_), were derived by fitting the concentration series using the manufacturer’s ImageAnalysis software (Version 2.4.7, Biosensing Instrument Inc., Tempe, AZ, USA), with double referencing (reference and blank subtraction) applied.

### 2.11. Allograft Tumor Model

Male C57BL/6 mice (6–8 weeks old, weighing approximately 20–25 g) were obtained from the Animal Experimental Center of Lanzhou University. Mice were housed under specific-pathogen-free (SPF) conditions with ad libitum access to food and water, a 12-h light/dark cycle, 22 ± 2 °C, and controlled humidity, in accordance with Lanzhou University Animal Experimental Center standards. To establish an allograft model, Hepa1-6-Luc cells were implanted at a density of 1–5 × 10^6^ per animal into the right axillary subcutaneous space of 6–8-week-old male mice. Gilteritinib was dissolved in a vehicle consisting of 2% DMSO, 5% Tween 80, and 93% saline. When tumors reached a measurable size (~7 days post-inoculation), mice were randomly assigned to control (vehicle) or gilteritinib (10 mg/kg, intragastric, every two days) groups (n = 6/group, total 12 mice). No a priori sample size calculation was performed; group sizes were based on previous comparable allograft studies in our laboratory. Body weights and tumor volumes of all mice were measured every 2 days throughout the experiment. Tumor volume was defined as the primary outcome measure. Tumor dimensions were monitored using calipers, and the volumes were calculated using the standard equation: V = (length × width^2^)/2. At the study endpoint, tumors were resected and evaluated. Mice were then humanely euthanized by carbon dioxide inhalation followed by cervical dislocation.

Tumor volume measurements and bioluminescence imaging were performed by an independent investigator blinded to group allocation. Blinding was not applied during drug administration or data analysis. No pre-specified inclusion or exclusion criteria were established. All mice completed the study and were included in the primary outcome (tumor volume) analysis; bioluminescence imaging and tumor histological analyses were performed on randomly selected subsets of mice (n = 3 per group), as detailed in the respective methods and figure legends. To minimize potential confounders, treatment allocation and measurements were performed in a randomized order, and cages were housed in equivalent positions in the animal facility.

### 2.12. Bioluminescence Imaging of Tumor Growth In Vivo

To longitudinally monitor tumor progression in living animals, bioluminescence imaging was performed using a bioluminescence/fluorescence imaging system (VOLHOO TRADE LIMITED, Hong Kong, China). Hepa1-6-Luc cells were sourced from Zeesan Biotechnology Co., Ltd., Wuhan, China. Hepa1-6-Luc cells stably express luciferase. Bioluminescent signals were captured at four time points (day 1, day 7, day 11, and day 13) after the initiation of treatment. Before imaging, each mouse received an intraperitoneal injection of D-luciferin (150 mg/kg dissolved in PBS). Ten minutes later, the animals were anesthetized with 2% isoflurane, and images were acquired. Bioluminescence intensity was quantified as mean flux (photons/s/cm^2^/sr) within a fixed region of interest (ROI) using Kuant software (Version 2.6.1, Vilber Lourmat, Eberhardzell, Germany).

### 2.13. Histological Analysis

Tumor tissues were fixed in 10% neutral buffered formalin for 24 h, embedded in paraffin, and sectioned at 4 μm. The sections were deparaffinized, rehydrated, and stained with hematoxylin and eosin (H&E). Representative photomicrographs of tumor sections were captured under a light microscope. Total tumor area and necrotic regions were manually delineated with the Freehand tool in ImageJ software. All image quantification was performed in a blinded manner. The percentage of tumor necrosis was calculated as total necrotic area divided by total tumor area × 100%.

### 2.14. Serum Biochemical Analysis

To evaluate the toxicity of gilteritinib, blood samples were collected from all mice (n = 6 per group) at the study endpoint prior to euthanasia. Blood was collected from the retro-orbital venous plexus using glass capillary tubes under 2% isoflurane anesthesia. The blood was allowed to clot at room temperature for 120 min and then centrifuged at 3000× *g* for 15 min at 4 °C to obtain serum. Serum was assessed using an automated biochemical analyzer to evaluate hepatic and renal functions, along with other serum chemistry markers.

### 2.15. Statistical Analysis

Experimental replicates and statistical unit: For all in vitro experiments (CCK-8, Western blot, Transwell, flow cytometry, total calpain activity, and wound healing assays), each data point (n = 3) represents an independent biological replicate from a separate cell culture experiment. Within each independent experiment, conditions were tested in technical triplicate, and technical replicates were averaged before statistical analysis. Statistical analyses were performed on the three independent biological replicates. For in vivo experiments (tumor growth, body weight, serum biochemistry), each mouse was treated as an independent biological replicate (n = 6 per group for tumor volume, body weight, and serum biochemistry; n = 3 per group for bioluminescence imaging and histological analysis). All data are presented as mean ± SD.

Statistical analyses were performed using GraphPad Prism software (Version 9.1.0, GraphPad Software, LLC, San Diego, CA, USA). Normality was assessed using the Shapiro–Wilk test, and homogeneity of variances was assessed using Levene’s test. Given the limited sample size (n = 3–6 per group), these tests have limited discriminatory power; however, no obvious deviations from normality or variance homogeneity were detected, and parametric tests were therefore applied. Body weight and bioluminescence intensity were analyzed by two-way repeated-measures ANOVA followed by Bonferroni’s post hoc test at each time point. For tumor growth data, linear mixed-effects models were used as the primary analysis to account for individual baseline variability, and two-way repeated-measures ANOVA followed by Bonferroni’s post hoc test was additionally applied as a complementary analysis; the two approaches yielded consistent results. For multiple independent group comparisons (in vitro assays with 0, 5, 10 μM, including Transwell, apoptosis, Western blot, and total calpain activity), one-way ANOVA with Dunnett’s post hoc test was used. For two-group comparisons, an unpaired two-tailed *t*-test was used. *p* < 0.05 was considered significant.

## 3. Results

### 3.1. Gilteritinib Reduces the Viability of HCC Cells

The effect of gilteritinib on the viability of Hepa1-6 and Huh-7 HCC cells was examined using the CCK-8 assay following exposure to increasing concentrations of gilteritinib. In Hepa1-6 cells, gilteritinib treatment resulted in a gradual decline in cell viability as the concentration increased (Figure 1A). Cell viability remained relatively stable at lower concentrations but decreased markedly above 10 μM, reaching very low levels at higher doses. Consistent with this observation, dose–response curve analysis showed that the IC_50_ of gilteritinib was 11.63 μM in Hepa1-6 cells (Figure 1B), indicating potent inhibition. Similarly, in Huh-7 cells, gilteritinib reduced cell viability in a concentration-dependent manner (Figure 1C). Although lower concentrations had limited effects, higher concentrations significantly reduced cell viability. The IC_50_ for Huh-7 cells was 13.59 μM (Figure 1D), confirming that human HCC cells are sensitive to gilteritinib treatment. Together, these findings indicate that gilteritinib effectively reduces the viability of both murine and human HCC cells.

Based on the IC_50_ values determined in the CCK-8 assays (11.63 μM for Hepa1-6 and 13.59 μM for Huh-7), 5 μM and 10 μM gilteritinib were selected for subsequent mechanistic experiments—migration, invasion, apoptosis, cell cycle, Western blot, and enzymatic activity assays. The 5 μM concentration corresponds to a sub-IC_50_ dose, whereas 10 μM is near-IC_50_. The 10 μM concentration partially but significantly reduced cell viability (~40–50% at 24 h), whereas 5 μM had more modest but still significant effects. These concentrations were deliberately selected to avoid extensive cell death, which would confound the interpretation of downstream signaling and molecular analyses, such as Western blot and enzymatic activity assays. The use of two concentrations also enabled us to assess dose-dependent effects on molecular endpoints, including calpain-1 expression, STAT3/AKT phosphorylation, and apoptosis-related proteins.

### 3.2. Gilteritinib Suppresses Wound Closure in HCC Cells

Wound healing assays were performed using Hepa1-6 and Huh-7 cells to determine whether gilteritinib affects the migratory capacity of HCC cells. In Hepa1-6 cells, control cells showed progressive wound closure over time, reflecting strong migratory ability (Figure 2A). In contrast, gilteritinib treatment markedly delayed wound closure, especially at the higher concentration. Quantitative analysis showed that the migration rate increased steadily in the control group, whereas both 5 μM and 10 μM gilteritinib significantly reduced migration, with the strongest inhibition at 10 μM (Figure 2B). Similarly, Huh-7 cells exhibited rapid wound closure under control conditions (Figure 2C). In contrast, gilteritinib substantially reduced cell migration across the wound area. Consistent with the imaging results, migration rate analysis showed a clear reduction in wound closure in treated cells relative to controls, and this inhibitory effect was more pronounced at the higher drug concentration (Figure 2D). Therefore, these findings indicate that gilteritinib impairs HCC cell migration, with the reduction in wound closure being most pronounced at the higher concentration.

### 3.3. Gilteritinib Inhibits Motility of HCC Cells in Transwell Assays

Transwell-based migration and invasion assays were performed in Hepa1-6 and Huh-7 cells to further examine the effects of gilteritinib on HCC cell motility. In the control group, cells migrated through the membrane (Figure 3A). In contrast, gilteritinib treatment markedly reduced the number of migrated cells in a concentration-dependent manner. Quantitative analysis showed that the number of migrated cells was significantly decreased in both the 5 μM and 10 μM groups compared with the control, with the strongest inhibition at 10 μM (Figure 3C). Consistent with the migration results, gilteritinib also suppressed the invasive capacity of Hepa1-6 cells. As shown in Figure 3B, fewer cells invaded through the Matrigel-coated membrane following gilteritinib treatment. Statistical analysis showed a significant reduction in the number of invading cells in treated groups relative to the control (Figure 3D). In Huh-7 cells, gilteritinib significantly inhibited cell migration. Representative images showed a marked decrease in migrated cells following gilteritinib treatment (Figure 3E), and quantitative analysis confirmed a significant reduction in the number of migrated cells (Figure 3G). Furthermore, invasion assays demonstrated that gilteritinib effectively suppressed the invasive potential of Huh-7 cells. The number of invading cells was markedly lower in the gilteritinib-treated groups compared with controls (Figure 3F), which was further supported by quantitative analysis (Figure 3H). We acknowledge that because gilteritinib reduces cell viability at higher concentrations, we cannot entirely exclude a contribution of reduced cell survival to the decreased migration and invasion observed at 10 μM. Nevertheless, the significant inhibition of motility at the sub-IC_50_ concentration of 5 μM, together with the serum-free wound-healing design, supports a direct effect on cell motility beyond simple cytotoxicity. Collectively, these findings indicate that gilteritinib impairs HCC cell motility in a concentration-dependent manner.

### 3.4. Gilteritinib Induces Apoptosis in HCC Cells

To explore whether gilteritinib’s inhibitory effect on HCC cells involves the induction of apoptosis, an Annexin V/PI double-staining assay combined with flow cytometric analysis was performed. In Hepa1-6 cells, gilteritinib treatment markedly increased the apoptotic fraction relative to the control (Figure 4A). The proportion of apoptotic cells increased progressively with increasing drug concentration. The apoptotic rate was significantly higher at 5 µM and 10 µM, with the greatest increase at 10 µM (Figure 4B). Similarly, in Huh-7 cells, gilteritinib promoted apoptosis in a concentration-dependent manner. Flow cytometric assessment showed a marked increase in the Annexin V^+^ fraction after gilteritinib treatment (Figure 4C). Consistent with these observations, statistical analysis demonstrated a significant increase in the apoptotic rate following gilteritinib treatment, particularly at the higher concentration (Figure 4D). Together, these results indicate that gilteritinib suppresses HCC cell survival by inducing apoptosis.

### 3.5. Gilteritinib Induces G2/M-Phase Cell Cycle Arrest in Hepa1-6 Cells

Cell cycle distribution was analyzed by flow cytometry. In Hepa1-6 cells, representative histograms revealed a clear alteration in cell cycle distribution following gilteritinib treatment (Figure 4E). Relative to the control, gilteritinib-treated cells exhibited an increased proportion of cells in the G2/M phase, accompanied by a corresponding reduction in the G0/G1 phase population. Quantitative analysis further demonstrated that gilteritinib elevated the G2/M fraction while reducing the G0/G1 fraction (Figure 4F). These changes became more pronounced at the higher concentration. Gilteritinib-induced G2/M accumulation is consistent with recent findings in other solid tumor models, where gilteritinib monotherapy increased the G2/M-phase fraction and downregulated cyclin B1 and CDK1 [29]. Moreover, the observed G2/M arrest in our study occurred concomitantly with the inhibition of STAT3 and AKT phosphorylation (Figure 8). Because STAT3 and AKT signaling pathways are well-established regulators of cell cycle progression and directly affect the expression of cyclins and CDKs, the suppression of these pathways by gilteritinib provides a mechanistic context for the observed cell cycle blockade. These findings indicate that gilteritinib inhibits Hepa1-6 cell proliferation, at least in part, by inducing G2/M-phase accumulation.

### 3.6. Whole-Cell SPR Demonstrates Concentration-Dependent Cellular Association of Gilteritinib with Hepa1-6 Cells

To investigate whether gilteritinib generates detectable association signals with HCC cells, whole-cell SPR analysis was performed using Hepa1-6 cells. Representative bright-field and SPR images depicted SPR signal responses on the immobilized cell monolayers, consistent with the cellular association of gilteritinib with Hepa1-6 cells (Figure 5A). Kinetic analysis revealed a well-defined affinity cluster, and quantitative fitting yielded an apparent equilibrium dissociation constant (*K*_D_ = 6.269 × 10^−7^ M) for the cellular association of gilteritinib with Hepa1-6 cells. The association rate constant (*k*_a_) was 1.229 × 10^3^ M^−1^·s^−1^, and the dissociation rate constant (*k*_d_) was 1.286 × 10^−3^ s^−1^ (Figure 5B,C). Figure 5D shows the response curves for the association of various concentrations of gilteritinib (1 nM, 10 nM, 100 nM, 1 μM, and 10 μM) with Hepa1-6 cells. Over time, the response values increased rapidly, indicating the association phase. Higher concentrations (10 μM) resulted in higher peak response values, reflecting a greater number of associated molecules. After reaching a plateau, the response values decreased during the dissociation phase, as the drug dissociated from the immobilized cells. The rate of decline in the curves reflects the speed of molecular dissociation. The concentration-dependent curve demonstrates that the association signals of gilteritinib with Hepa1-6 cells increased progressively with increasing drug concentrations (Figure 5E).

### 3.7. Whole-Cell SPR Demonstrates Concentration-Dependent Cellular Association of Gilteritinib with Huh-7 Cells

Whole-cell SPR analysis was similarly performed using Huh-7 cells. Bright-field and SPR images revealed localized signal responses on the immobilized cell monolayers (Figure 6A). Kinetic parameter distribution analysis revealed a distinct affinity cluster (Figure 6B), and quantitative fitting yielded an apparent *K*_D_ of 4.259 × 10^−7^ M (*k*_a_ = 5.004 × 10^3^ M^−1^·s^−1^ and *k*_d_ = 1.796 × 10^−3^ s^−1^) (Figure 6C). The response curves for the cellular association of various concentrations of gilteritinib (1 nM, 10 nM, 100 nM, 1 μM, and 10 μM) with Huh-7 cells were consistent with those observed in Hepa1-6 cells (Figure 6D). Over time, the response values increased rapidly, and higher concentrations resulted in higher peak response values, exhibiting a concentration-dependent relationship (Figure 6E). These findings suggest that gilteritinib shows concentration-dependent whole-cell association with both Hepa1-6 and Huh-7 cells in SPR analyses, providing evidence of cellular engagement that supports its subsequent intracellular pharmacological effects.

### 3.8. Gilteritinib Suppresses Hepa1-6 Tumor Growth In Vivo

To evaluate the antitumor effect of gilteritinib in vivo, a subcutaneous allograft tumor model was established using Hepa1-6-Luc cells, and male C57BL/6 mice were treated with gilteritinib (10 mg/kg). At the experimental endpoint, tumors excised from the gilteritinib-treated group were visibly smaller than those from the control group (Figure 7A). Quantitative analysis confirmed that tumor volume derived from Hepa1-6 cells was significantly reduced following gilteritinib treatment (Figure 7B). Consistently, longitudinal measurements demonstrated that tumor growth was markedly inhibited in the gilteritinib-treated group, with significant differences emerging at later time points (Figure 7C). Throughout the treatment period, body weight remained stable in both groups, with no inter-group differences observed at any measured time point. General behavior, food intake, and activity levels were also comparable between control and gilteritinib-treated mice (Figure 7D). Bioluminescence imaging further revealed progressive signal enhancement in control mice, whereas bioluminescence signals remained substantially lower in mice bearing Hepa1-6 tumors treated with gilteritinib (Figure 7E). Quantitative analysis of bioluminescence intensity showed no significant difference at early time points but demonstrated a significant reduction at later stages in the gilteritinib-treated group (Figure 7F). To further evaluate the in vivo antitumor effect of gilteritinib, H&E-stained subcutaneous Hepa1-6 tumor sections were analyzed. Representative histological images are shown in Figure 7G. Blinded quantitative analysis demonstrated that the percentage of tumor necrosis was significantly higher in the gilteritinib-treated group (17.18 ± 4.25%) compared with vehicle control (8.56 ± 2.37%; unpaired *t*-test, *p* = 0.037) (Figure 7H). Accordingly, the proportion of viable tumor tissue was reduced in the gilteritinib 10 mg/kg group. These findings indicate that gilteritinib inhibited tumor growth under the current experimental conditions.

### 3.9. Gilteritinib Treatment Is Associated with Reduced STAT3/AKT Phosphorylation and Altered Apoptosis-Related Protein Expression

Western blotting was performed to assess the levels of key proteins involved in signal transduction and apoptosis and to clarify how gilteritinib exerts its anticancer activity at the molecular level. In Hepa1-6 cells, gilteritinib treatment did not affect total STAT3 or AKT protein levels but significantly reduced their phosphorylation in a concentration-dependent manner. Bcl-2 expression decreased after gilteritinib treatment (Figure 8A,C). In tumor tissues, gilteritinib also reduced STAT3 and AKT phosphorylation without affecting total protein levels (Figure 8B,D). Consistently, gilteritinib downregulated Bcl-2 and upregulated Bax (Figure 8E). These results indicate that gilteritinib inhibits the STAT3/AKT pathway and induces apoptosis both in vitro and in vivo.

**Figure 8:**
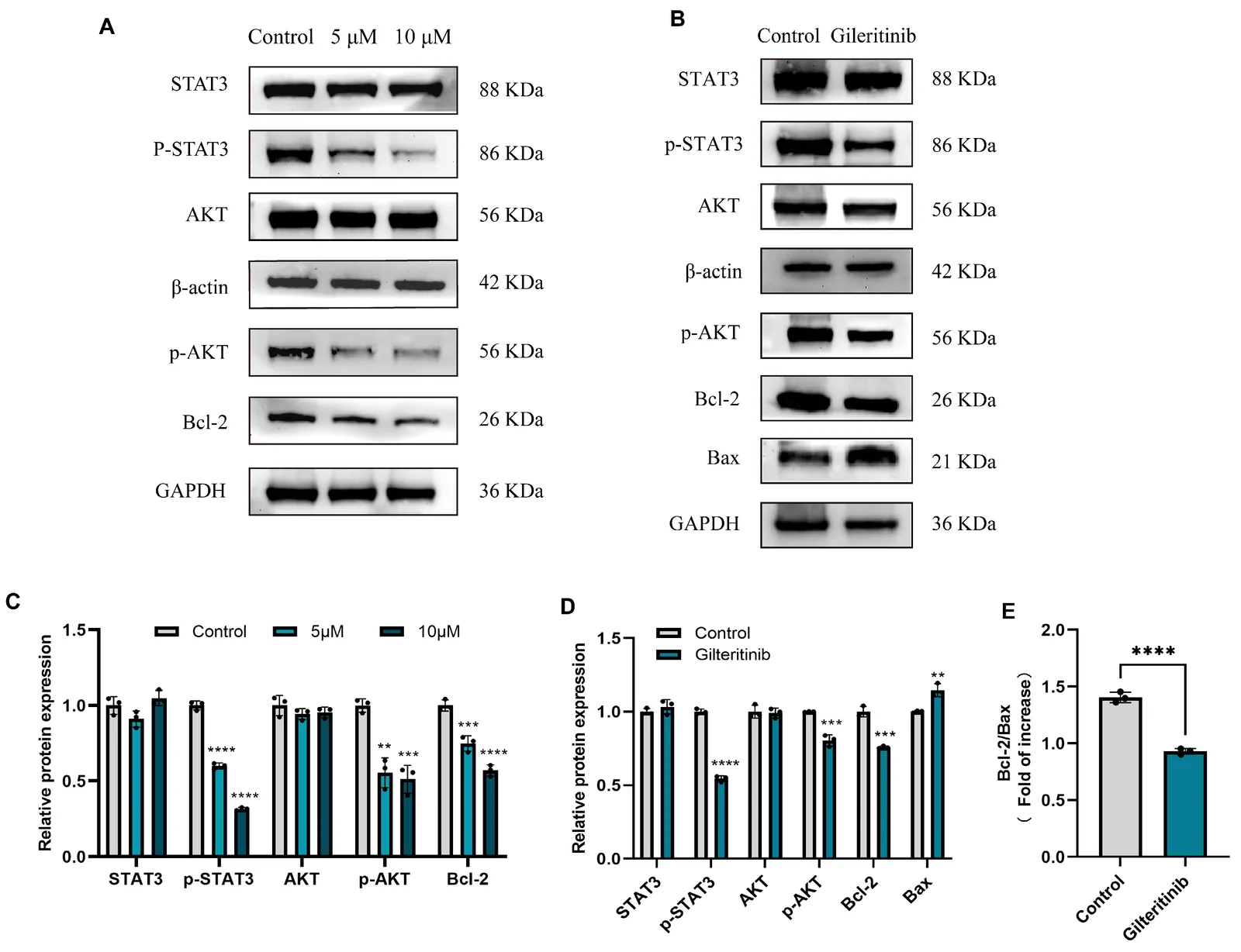
Gilteritinib inhibits STAT3/AKT signaling and regulates apoptosis-related proteins in Hepa1-6 cells and allograft tumors. (**A**) Representative immunoblots depict the expression of STAT3, p-STAT3, AKT, p-AKT, and Bcl-2 in Hepa1-6 cells treated with gilteritinib (0, 5, 10 μM) for 24 h. (**B**) Representative immunoblots of STAT3, p-STAT3, AKT, p-AKT, Bcl-2, and Bax in Hepa1-6 allograft tumor tissues (control vs. gilteritinib 10 mg/kg). (**C**) Densitometric quantification of protein levels in Hepa1-6 cells. (**D**) Quantification of protein levels in tumor tissues. (**E**) Quantification of the Bcl-2/Bax ratio in tumor tissues. β-actin and GAPDH were used as internal loading controls. Data are presented as mean ± SD. n = 3 represents three independent biological replicates. For three-group comparisons (**C**), one-way ANOVA with Dunnett’s post hoc test was applied; for two-group comparisons (**D**,**E**), unpaired two-tailed *t*-test was used. ** *p* < 0.01, *** *p* < 0.001, **** *p* < 0.0001 versus the control group.

### 3.10. Gilteritinib Reduces Calpain-1 Expression and Total Calpain Activity in Hepa1-6 Cells and Allograft Tumors

In Hepa1-6 cells, Western blot analysis showed that gilteritinib treatment markedly reduced calpain-1 protein expression in a concentration-dependent manner (Figure 9A,B). Consistently, total calpain activity was significantly decreased following gilteritinib treatment relative to the control (Figure 9C). To further validate these findings in vivo, tumor tissues from Hepa1-6 allografts were examined. As shown in Figure 9D,E, gilteritinib treatment significantly decreased calpain-1 protein levels in tumor tissues. In agreement with this result, total calpain activity was also significantly reduced in the gilteritinib-treated group (Figure 9F). These findings indicate that gilteritinib inhibits both calpain-1 expression and total calpain activity in HCC cells and tumors.

### 3.11. Gilteritinib Does Not Cause Significant Alterations in Serum Biochemical Parameters in the Hepa1-6 Tumor Model

Serum biochemical parameters were measured in Hepa1-6 tumor-bearing mice after treatment to assess the safety of gilteritinib in vivo (Figure 10). The control and gilteritinib-treated groups showed comparable levels of all tested liver function parameters, including AST, ALT, ALP, ALB, A/G ratio, TB, and TBA. Renal function-related markers (CRE, UREA, and UA) were also comparable between groups. In addition, other biochemical parameters, including CHE, CK, CO_2_, GLB, and LDH, showed no significant changes following gilteritinib administration. Taken together, these findings indicate that gilteritinib had no overt toxic effects on serum biochemical markers under the current treatment schedule.

## 4. Discussion

In the present study, we found that the antitumor effects of gilteritinib in HCC correlate with reduced calpain-1 expression and inhibition of STAT3/AKT signaling. Using both murine Hepa1-6 and human Huh-7 HCC cell models, our data demonstrated that gilteritinib reduced HCC cell proliferation, motility, and invasion, triggered cell cycle arrest and apoptosis, and suppressed tumor growth in vivo. Mechanistically, gilteritinib reduced calpain-1 expression and total calpain activity, decreased STAT3 and AKT phosphorylation, and altered the expression of apoptosis-related proteins, including downregulation of Bcl-2 and upregulation of Bax. Furthermore, SPR analysis confirmed that gilteritinib exhibits concentration-dependent whole-cell association with both Hepa1-6 and Huh-7 cells. Importantly, gilteritinib effectively inhibited tumor growth in Hepa1-6 tumor-bearing mice without overt toxicity in the limited parameters evaluated, supporting its therapeutic potential and safety. It should be noted that our findings show associations rather than causal relationships. The reduction in calpain-1 expression and total calpain activity correlates with inhibition of STAT3/AKT signaling and antitumor effects, but causal evidence requires future genetic manipulation studies.

Calpain-1 has emerged as a critical regulator of tumor progression, participating in diverse cellular processes. In the present study, we found that gilteritinib treatment significantly reduced calpain-1 expression and total calpain activity in both cultured cells and tumor tissues. Given calpain-1’s established roles in regulating STAT3 and AKT signaling, we next examined whether these pathways were affected. Notably, calpain-1 reduction was accompanied by decreased STAT3 and AKT phosphorylation, while total protein levels remained unchanged, suggesting that gilteritinib primarily affects signaling activation rather than protein expression. This distinction is important because phosphorylation-dependent activation of STAT3 and AKT is a key driver of tumor progression [30]. The downstream effects of pathway inhibition were further supported by changes in apoptosis-related proteins, including decreased expression of Bcl-2 and increased expression of Bax. These molecular changes were consistent with our functional findings showing increased apoptosis and G2/M-phase cell cycle arrest following gilteritinib treatment. Together, these results are consistent with the hypothesis that gilteritinib treatment is associated with reduced calpain-1 expression and total calpain activity, in parallel with suppression of STAT3/AKT phosphorylation and activation of apoptotic pathways. However, whether these events are causally linked, and the precise direction of this relationship, remain to be established.

Gilteritinib is a multi-kinase inhibitor that also targets FLT3, AXL, ALK, and LTK. Its antitumor activity therefore likely involves the simultaneous modulation of multiple targets and pathways, with calpain-1 appearing to be one important node. Accordingly, the suppression of STAT3/AKT phosphorylation observed here may reflect both direct inhibition of upstream receptor kinases and downstream consequences of calpain-1 downregulation. Consistent with this view, calpain has been reported to regulate STAT3 phosphorylation through a Ca^2+^/calpain-dependent axis [31]. Calpain activation can also inhibit AKT signaling, for instance, through activation of the tumor suppressor PTEN [32]. These observations suggest that calpain-1 likely acts in conjunction with other signaling molecules rather than in isolation.

Our findings also showed that gilteritinib significantly inhibited migration and invasion of HCC cells. Tumor metastasis is a major cause of poor prognosis in HCC, and calpain-1 has been implicated in the regulation of cytoskeletal dynamics and cell motility. This is supported by evidence that calpain-1 acts as a primary protease that cleaves the ectodomain of hepsin, a transmembrane serine protease involved in tumor progression, on hepatoma cell surfaces [33]. Therefore, the observed reduction in migration and invasion may partly reflect reduced calpain-1 expression, although a causal role for calpain-1 in this process remains to be demonstrated.

Beyond its mechanistic relevance, calpain-1 may also be a promising biomarker in HCC. Elevated expression of CAPNS1 (Capn4), the common regulatory subunit of calpain-1/2, has been associated with shorter overall survival across multiple cancer types [34], including liver cancer [14]. Our finding that gilteritinib consistently reduces calpain-1 expression and total calpain activity in vitro and in vivo, in parallel with antitumor efficacy, suggests that calpain-1 could serve as a candidate pharmacodynamic or prognostic biomarker. However, establishing calpain-1 as a robust biomarker will require further investigation. Specifically, future studies could include (1) correlating tumoral calpain-1 expression with patient outcomes in HCC cohorts; (2) longitudinal monitoring of calpain-1 activity in peripheral blood or tumor biopsies during gilteritinib treatment; and (3) performing calpain-1 knockdown/rescue experiments to confirm its functional contribution to gilteritinib sensitivity.

Concentration-dependent whole-cell association of gilteritinib with HCC cells was shown by SPR analysis in both Hepa1-6 and Huh-7 cells, yielding apparent equilibrium dissociation constants (*K*_D_) of 6.269 × 10^−7^ M and 4.259 × 10^−7^ M, respectively. These data indicate that gilteritinib can associate with intact HCC cells in a concentration-dependent and saturable manner, providing evidence of drug engagement at the cellular level. This association is consistent with the subsequent intracellular effects observed, including reduced calpain-1 expression and inhibited STAT3/AKT phosphorylation. Although whole-cell SPR does not pinpoint the precise molecular target, the combination of SPR evidence (cellular engagement), Western blot findings (calpain-1 and STAT3/AKT modulation), and functional assays (reduced proliferation, migration, and increased apoptosis) forms a coherent correlative dataset supporting the hypothesis that gilteritinib engages HCC cells and modulates downstream signaling pathways associated with antitumor effects.

Importantly, the in vivo experiments using the Hepa1-6 allograft model demonstrated that gilteritinib significantly inhibited tumor growth, as shown by reduced tumor volume and decreased bioluminescence signals. These findings support the translational relevance of our in vitro observations. Moreover, serum biochemical analysis showed no significant changes in liver or kidney function markers, and body weight remained stable in both groups, with no differences between the two groups at any measured time point. No overt toxicity was detected using the biochemical and body weight parameters assessed under the present experimental conditions.

Despite these promising findings, several limitations should be acknowledged. First, although our data suggest a correlation between calpain-1 downregulation and the antitumor effects of gilteritinib, this study does not establish causation. The present study only shows that calpain-1 downregulation is accompanied by suppressed STAT3/AKT phosphorylation but does not establish that calpain-1 directly regulates these pathways or cooperates with other signaling molecules. Future investigations using genetic manipulation, such as calpain-1 knockdown or overexpression, are needed to confirm its causal role and delineate the interplay between calpain-1 and STAT3/AKT signaling. Second, SPR was performed on whole cells and does not identify the direct molecular target. Therefore, while these results suggest a potential cellular association, they do not demonstrate binding to a defined target protein. Future studies using target-identification approaches, such as cellular thermal shift assays (CETSA), drug-affinity responsive target stability (DARTS), or SPR using purified calpain-1 protein, are needed to identify the precise molecular target(s) responsible for the observed antitumor activity. Third, our in vivo safety evaluation was limited to serum biochemistry and body weight monitoring. Histopathological examination of liver, kidney, and spleen was not performed in the present study, which restricts our ability to fully characterize gilteritinib’s systemic toxicity profile. In addition, a direct comparison of gilteritinib’s effects between HCC cells and normal hepatic cells or primary hepatocytes (IC_50_, apoptosis, cell cycle, calpain-1 expression, and STAT3/AKT phosphorylation) was not performed, which limits the direct assessment of tumor selectivity; such comparisons represent an important direction for future investigation. Fourth, liver cancer-related biomarkers were not assessed, and the subcutaneous allograft model does not fully recapitulate the hepatic microenvironment of orthotopic HCC. Future studies using an orthotopic model with dynamic monitoring of liver cancer-related biomarkers would more closely mimic the hepatic microenvironment and further evaluate the antitumor efficacy of gilteritinib. Fifth, although flow cytometric analysis demonstrated G2/M cell-cycle accumulation, the molecular regulators of the G2/M transition (cyclin B1, CDK1, CDC25C, and checkpoint kinases) were not analyzed. Detailed molecular validation of cell-cycle effects is needed. Sixth, although the molecular changes (reduced calpain-1 expression, decreased STAT3/AKT phosphorylation, and altered Bcl-2/Bax) were consistently observed in both cultured HCC cells and whole-tumor lysates, immunohistochemistry or immunofluorescence was not performed to localize calpain-1, p-STAT3, and p-AKT within the tumor tissue or to correlate their changes with proliferation (Ki-67) and apoptosis. We acknowledge this limitation. Future studies should employ immunohistochemical and immunofluorescence analyses to evaluate the spatial distribution and cellular localization of these molecular changes in tumor tissues and to correlate them with proliferation and apoptosis markers, thereby providing a more comprehensive understanding of gilteritinib’s mechanism of action in vivo. Finally, additional studies are required to evaluate the effects of gilteritinib in other HCC models and in clinical samples.

## 5. Conclusions

In conclusion, this study demonstrates that gilteritinib exhibits antitumor activity against HCC in vitro and in vivo, correlating with downregulation of calpain-1, inhibition of STAT3/AKT signaling, and induction of apoptosis. These findings suggest that calpain-1 may represent a candidate downstream correlate of gilteritinib’s effects; however, direct targeting of calpain-1 by gilteritinib requires formal confirmation. Our results provide a rationale for further investigation of gilteritinib in HCC and highlight calpain-1 as a potential biomarker or therapeutic correlate worthy of future mechanistic exploration.

## Figures and Tables

**Figure 1:**
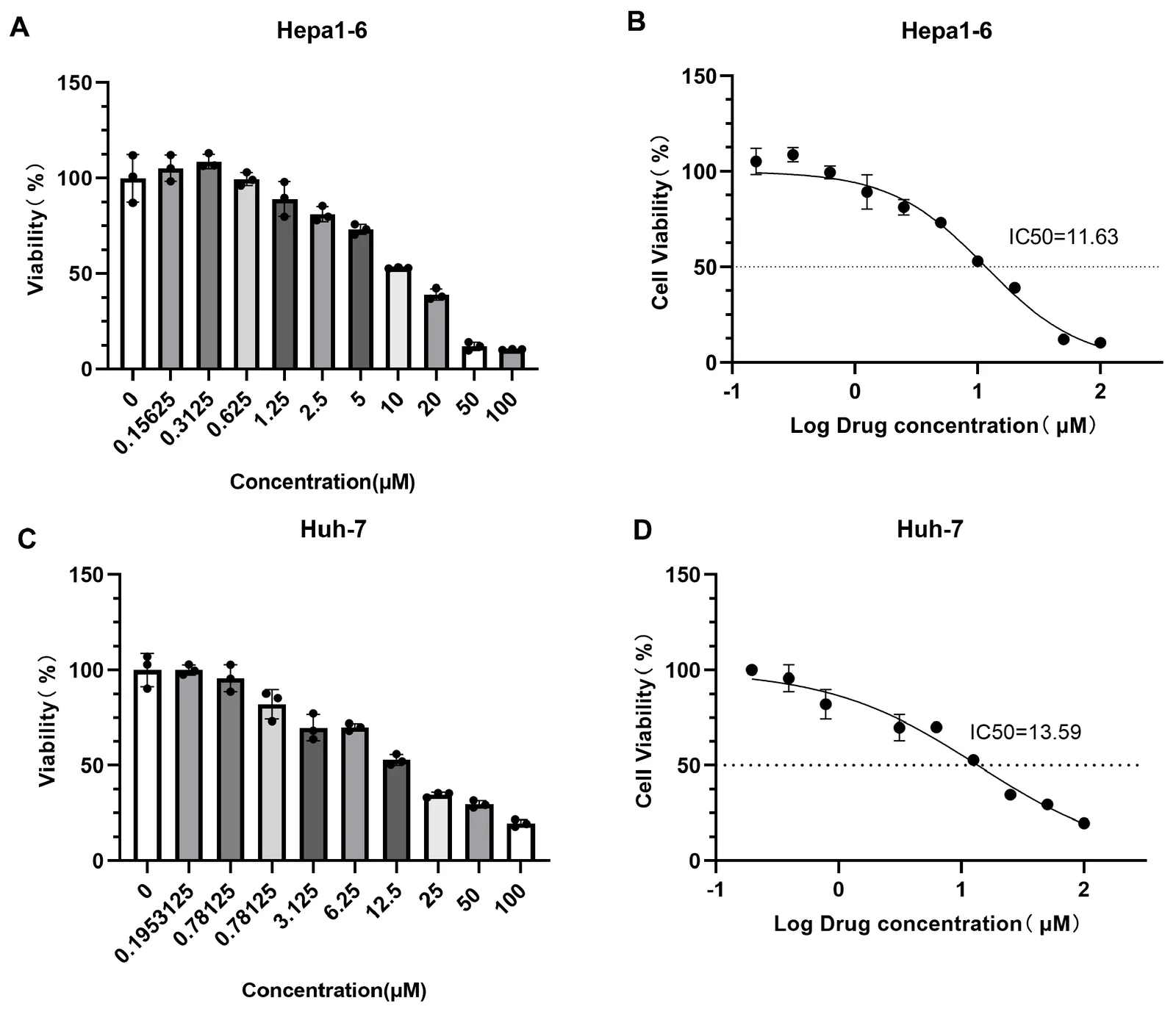
Gilteritinib reduces Hepa1-6 and Huh-7 HCC cell viability. (**A**) Effect of gilteritinib on Hepa1-6 cell viability measured using the CCK-8 assay after 24 h. (**B**) Dose–response curve and IC_50_ determination in Hepa1-6 cells. (**C**) Effect of gilteritinib on Huh-7 cell viability measured using the CCK-8 assay after 24 h. (**D**) Dose–response curve and IC_50_ determination in Huh-7 cells. n = 3 independent biological replicates. Data are presented as the mean ± SD.

**Figure 2:**
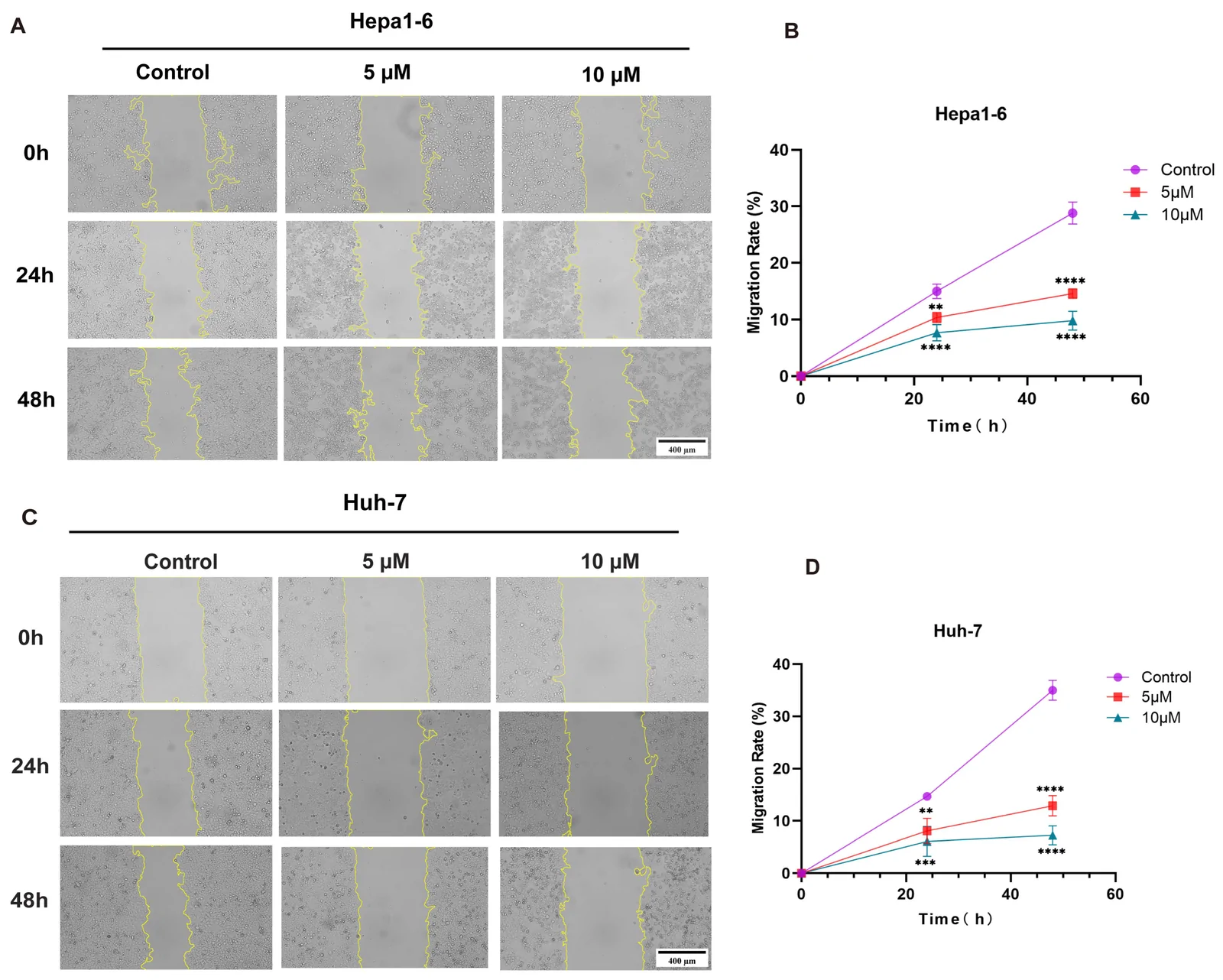
Gilteritinib inhibits wound closure in HCC cells. (**A**) Representative images of the wound healing assay in Hepa1-6 cells treated with gilteritinib (0, 5, and 10 μM) at 0, 24, and 48 h. (**B**) Quantification of migration rates in Hepa1-6 cells. (**C**) Representative wound healing images of Huh-7 cells treated with gilteritinib (0, 5, and 10 μM) at 0, 24, and 48 h. (**D**) Quantification of migration rates in Huh-7 cells. Scale bars: 400 µm. n = 3 independent biological replicates. Data were analyzed using two-way repeated-measures ANOVA (time: repeated measures; group: between-subject) followed by Dunnett’s post hoc test for multiple comparisons versus the control group. ** *p* < 0.01, *** *p* < 0.001, **** *p* < 0.0001.

**Figure 3:**
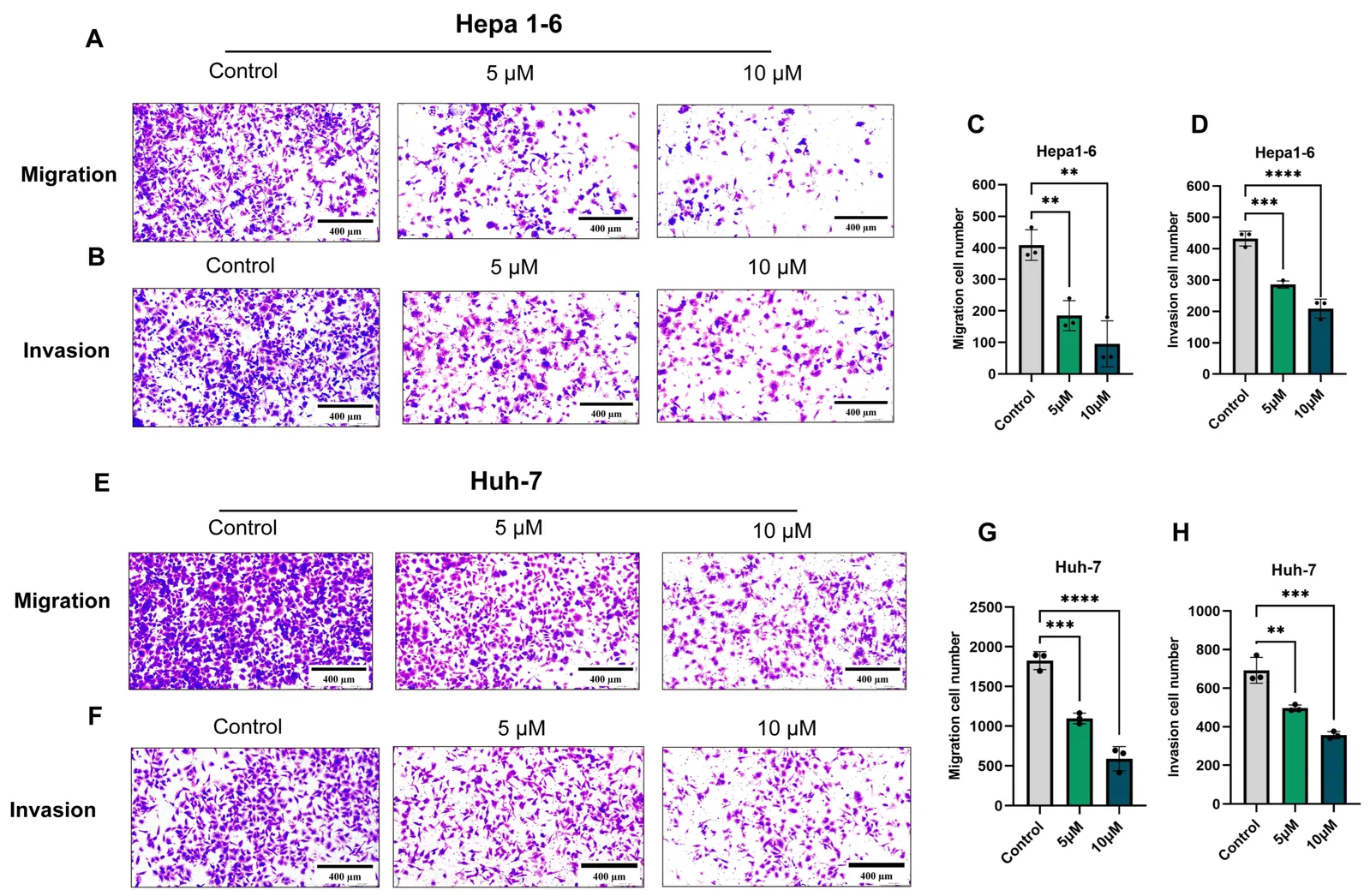
Gilteritinib inhibits motility of Hepa1-6 and Huh-7 HCC cells. (**A**) Representative images of Transwell migration assay in Hepa1-6 cells. (**B**) Representative images of Transwell invasion assay in Hepa1-6 cells. (**C**) Quantification of migrated Hepa1-6 cells. (**D**) Quantification of invaded Hepa1-6 cells. (**E**) Representative images of Transwell migration assay in Huh-7 cells. (**F**) Representative images of Transwell invasion assay in Huh-7 cells. (**G**) Quantification of migrated Huh-7 cells. (**H**) Quantification of invaded Huh-7 cells. Data are presented as mean ± SD. Data were analyzed by one-way ANOVA followed by Dunnett’s post hoc test for multiple comparisons against the control group. n = 3 represents three independent biological replicates. ** *p* < 0.01; *** *p* < 0.001; **** *p* < 0.0001. Scale bars: 400 µm.

**Figure 4:**
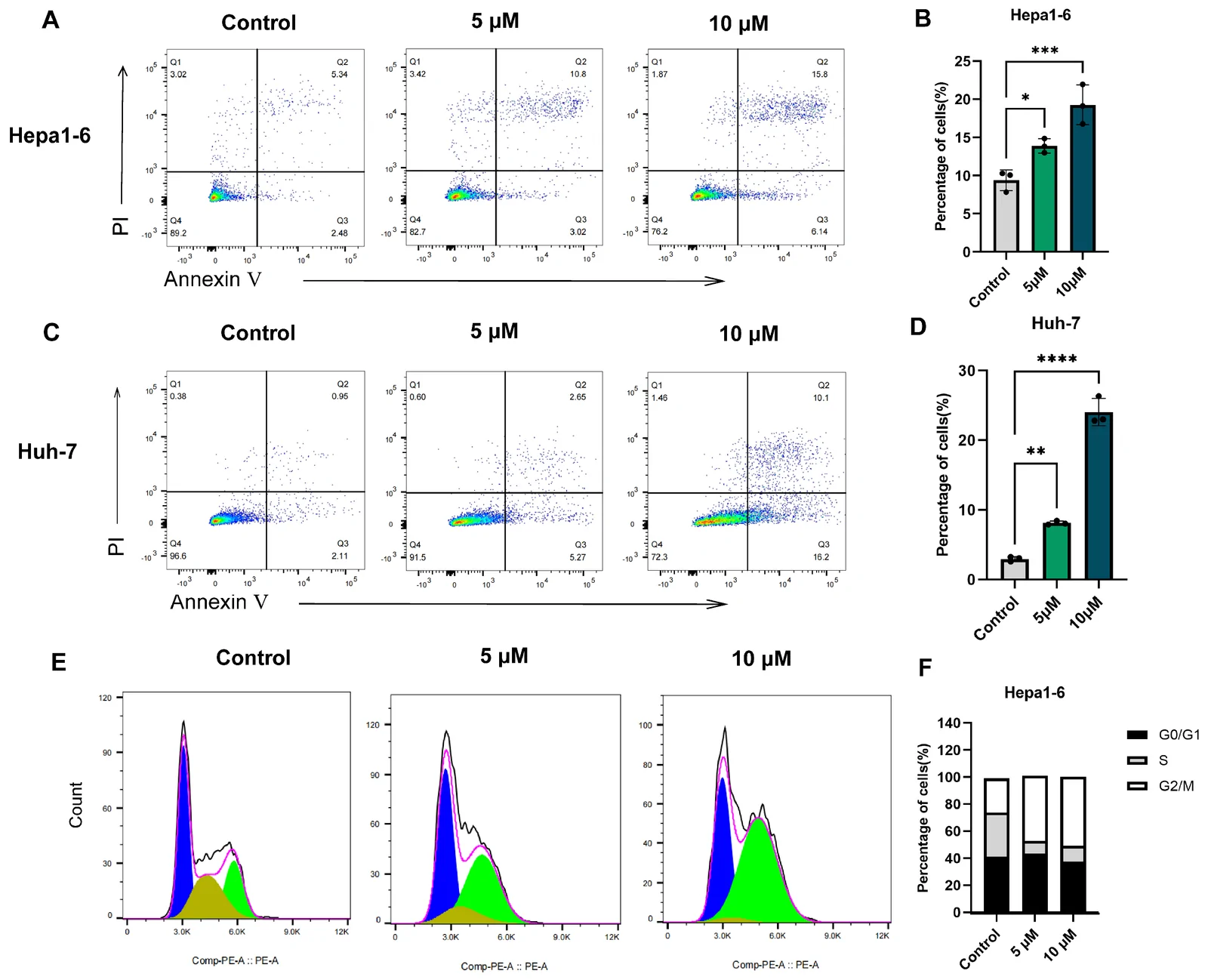
Gilteritinib induces apoptosis and is associated with G2/M-phase accumulation in HCC cells. (**A**) Representative flow cytometry plots of Annexin V/PI staining in Hepa1-6 cells treated with gilteritinib (0, 5, and 10 μM). (**B**) Quantitative analysis of apoptotic Hepa1-6 cells. (**C**) Representative flow cytometry plots in Huh-7 cells (0, 5, and 10 μM). (**D**) Quantification of apoptotic Huh-7 cells. (**E**) Representative flow cytometry histograms showing cell cycle distribution following gilteritinib treatment (0, 5, and 10 μM). (**F**) Quantitative analysis of cell cycle phase distribution. Data were analyzed by one-way ANOVA followed by Dunnett’s post hoc test for multiple comparisons against the control group. n = 3 represents three independent biological replicates. * *p* < 0.05; ** *p* < 0.01; *** *p* < 0.001; **** *p* < 0.0001.

**Figure 5:**
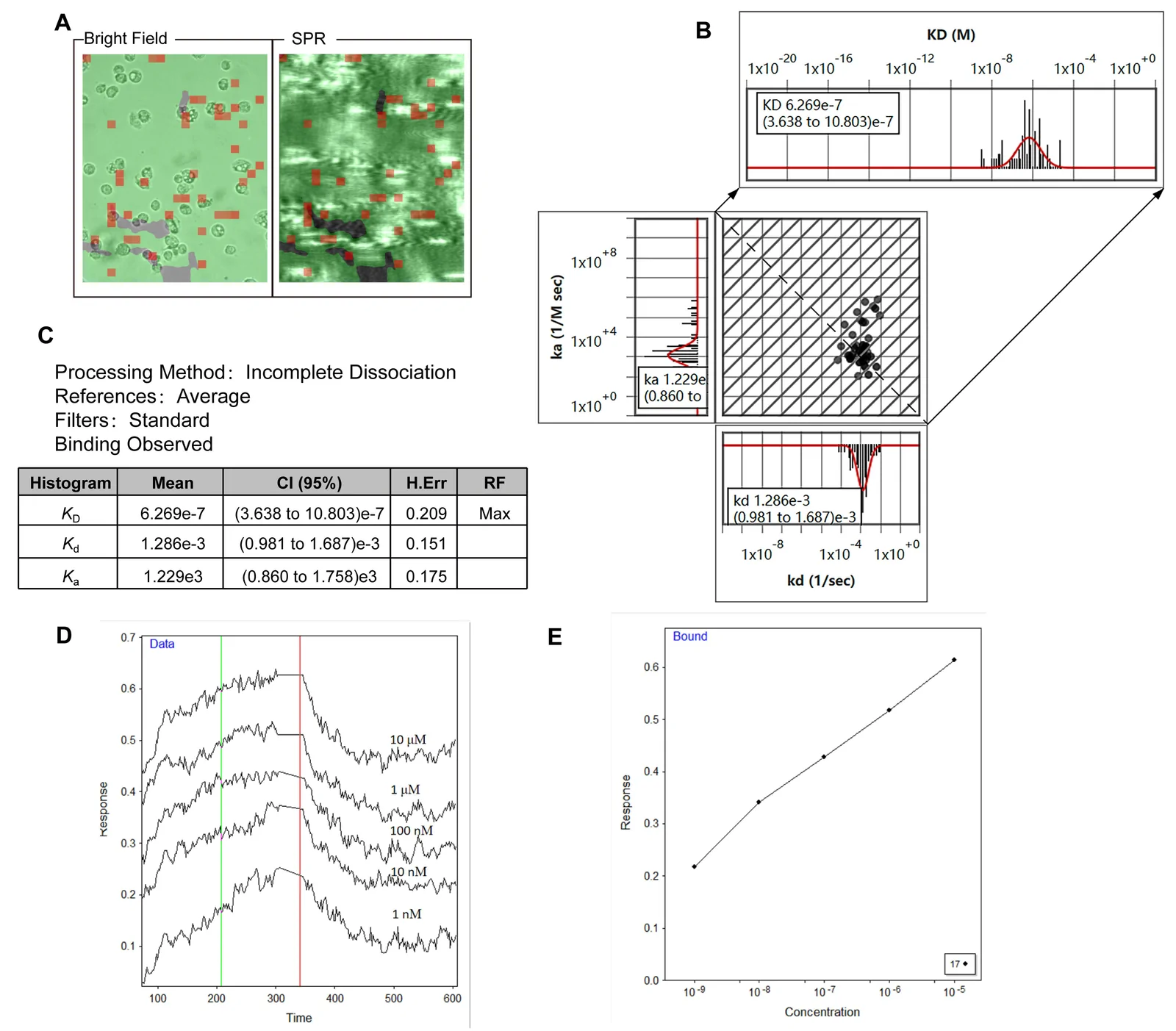
Gilteritinib associates with Hepa1-6 cells in a concentration-dependent manner. (**A**) Representative bright-field and SPR images depicting association signals. (**B**) Kinetic analysis and affinity distribution plot. (**C**) Summary of kinetic parameters, including apparent *K*_D_, *k*_a_, and *k*_d_. (**D**) Equilibrium response curves showing the response values detected for the association of various concentrations of gilteritinib (1 nM, 10 nM, 100 nM, 1 μM and 10 μM) with the Hepa1-6 cells. (**E**) Concentration-dependent curve demonstrating the enhancement of association signals with increasing concentrations of gilteritinib.

**Figure 6:**
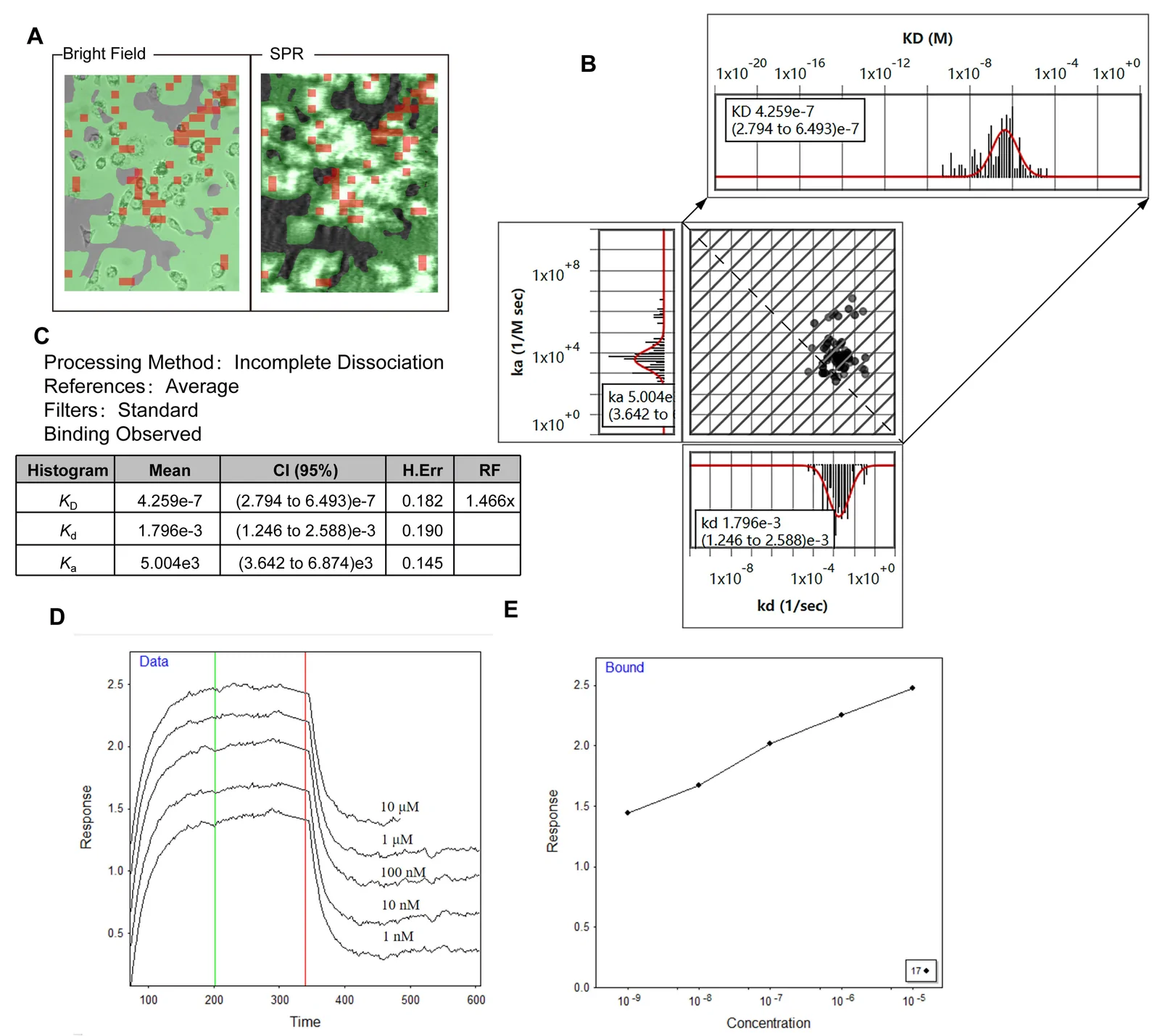
SPR analysis revealing whole-cell association signals between gilteritinib and Huh-7 cells. (**A**) Representative bright-field and SPR images. (**B**) Kinetic analysis and affinity distribution plot. (**C**) Summary of kinetic parameters (apparent *K*_D_, *k*_a_, *k*_d_). (**D**) Equilibrium response curves depicting the response values detected for the cellular association of various concentrations of gilteritinib (1 nM, 10 nM, 100 nM, 1 μM and 10 μM) with the Huh-7 cells. (**E**) The relationship curve depicting the concentration-dependent enhancement of association signals with increasing gilteritinib concentrations.

**Figure 7:**
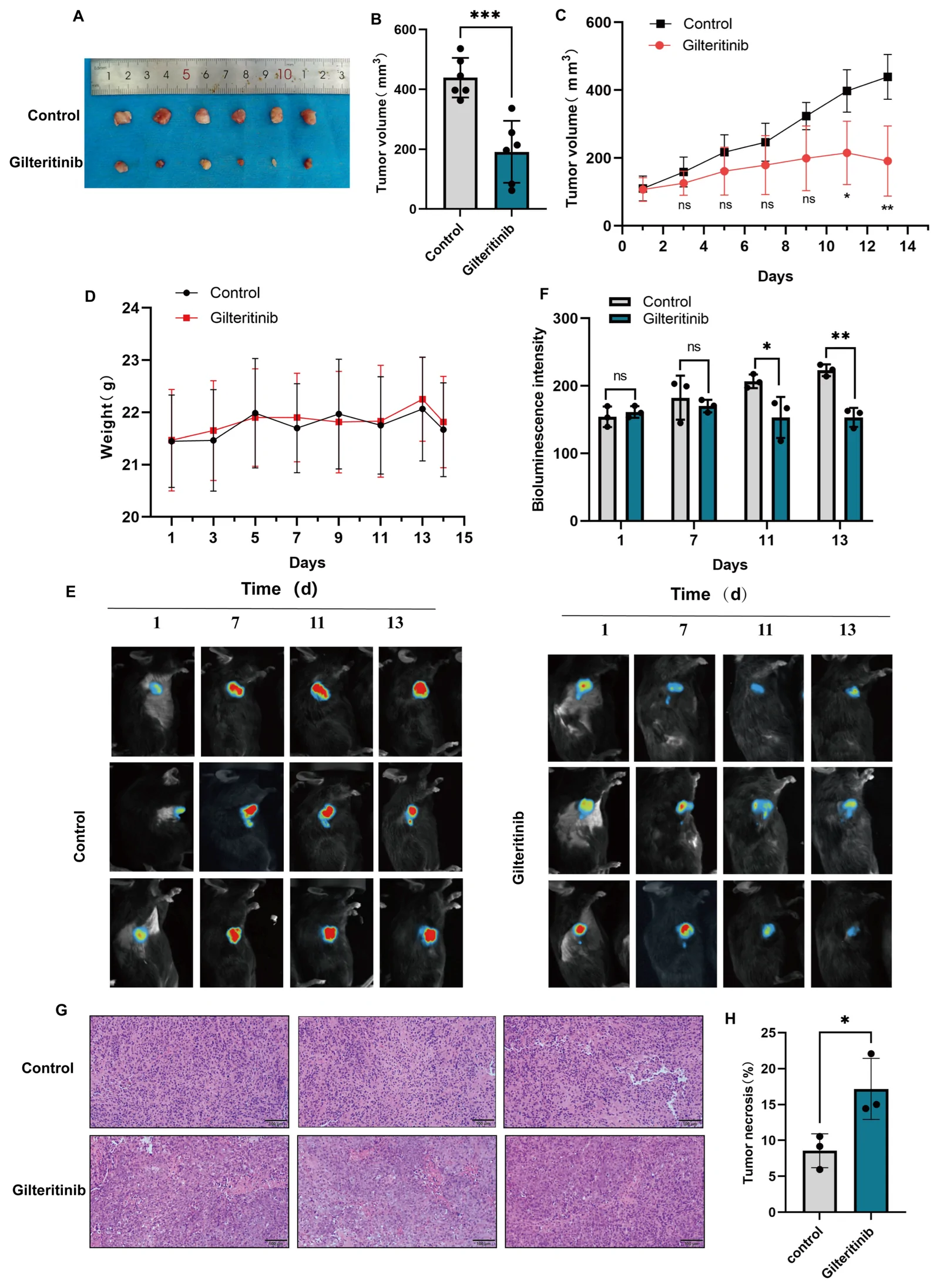
Gilteritinib inhibits Hepa1-6 allograft tumor growth in vivo. (**A**) Representative images of excised tumors derived from Hepa1-6 cells. (**B**) Tumor volume quantification. (**C**) Tumor growth curves. (**D**) Body weight changes in control and gilteritinib-treated mice. (**E**) Representative bioluminescence images of mice bearing Hepa1-6 tumors. (**F**) Quantification of bioluminescence intensity. (**G**) Representative H&E-stained tumor sections from vehicle control and gilteritinib-treated mice; scale bar, 100 μm. (**H**) Quantification of the necrotic area fraction. Endpoint tumor volume (**B**) and the necrotic area fraction (**H**) were compared using unpaired two-tailed *t*-test. Tumor growth curves (**C**) were analyzed using a linear mixed-effects model, with two-way repeated-measures ANOVA (time: repeated measures; group: between-subject) followed by Bonferroni’s post hoc test at each time point as a complementary analysis. Body weight (**D**) and bioluminescence intensity (**F**) were analyzed by two-way repeated-measures ANOVA followed by Bonferroni’s post hoc test at each time point. n = 6 represents six individual mice per group for (**B**–**D**); n = 3 mice represents three individual mice per group for (**E**–**H**). ns, not significant; * *p* < 0.05, ** *p* < 0.01, *** *p* < 0.001.

**Figure 9:**
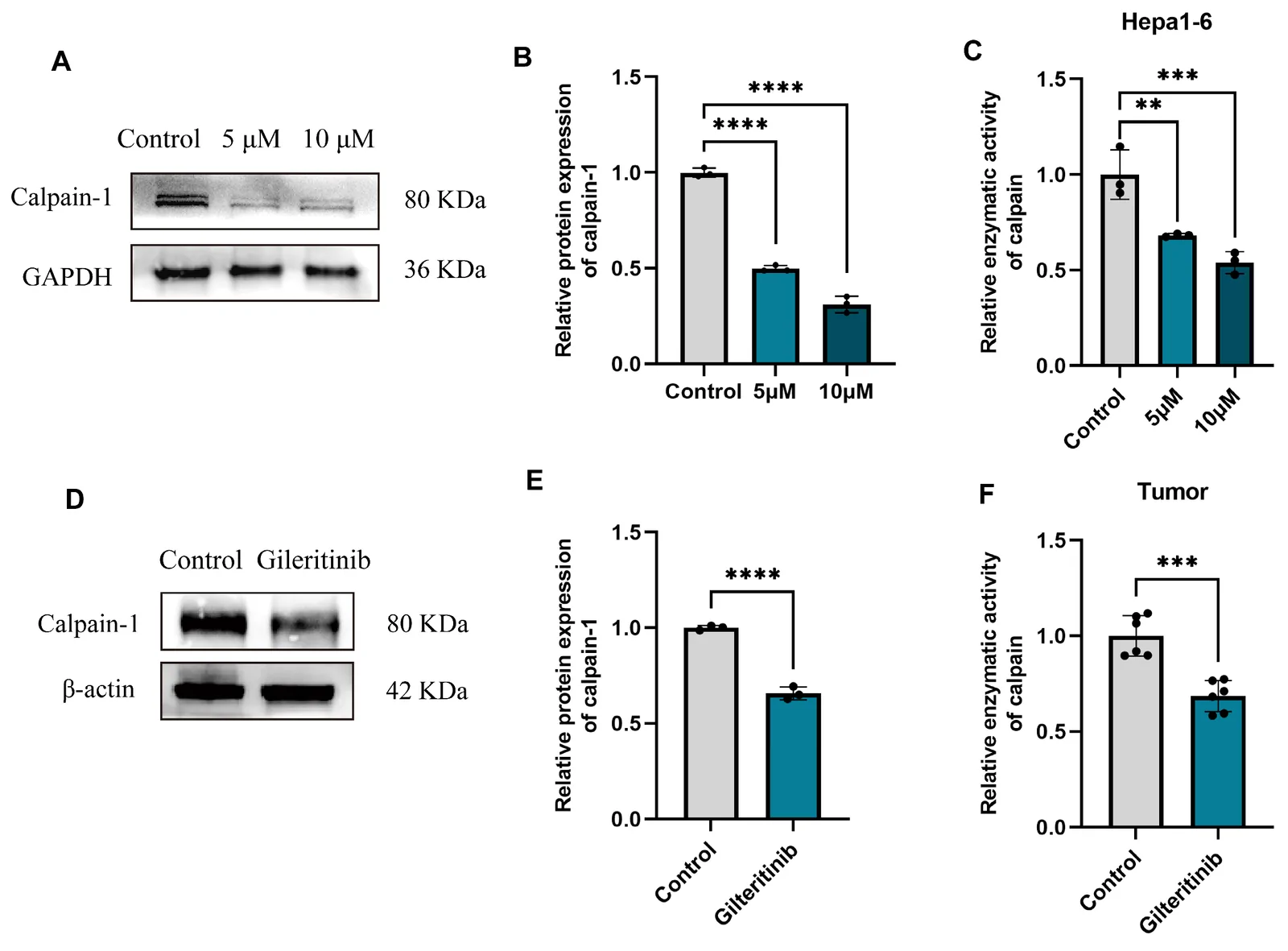
Gilteritinib inhibits calpain-1 expression and total calpain activity in Hepa1-6 cells and allograft tumors. (**A**) Western blot analysis of calpain-1 expression in Hepa1-6 cells treated with gilteritinib (0, 5 and 10 μM). (**B**) Densitometric quantification of calpain-1 protein expression in Hepa1-6 cells. (**C**) Quantification of total calpain activity in Hepa1-6 cells. (**D**) Western blot analysis of calpain-1 expression in Hepa1-6 allograft tumor tissues. (**E**) Quantification of calpain-1 protein expression in tumor tissues. (**F**) Quantification of total calpain activity in tumor tissues. Data are mean ± SD (n = 3 represents three independent biological replicates). For three-group comparisons (**B**,**C**), one-way ANOVA with Dunnett’s post hoc test was applied; for two-group comparisons (**E**,**F**), unpaired two-tailed *t*-test was used. ** *p* < 0.01; *** *p* < 0.001; **** *p* < 0.0001.

**Figure 10:**
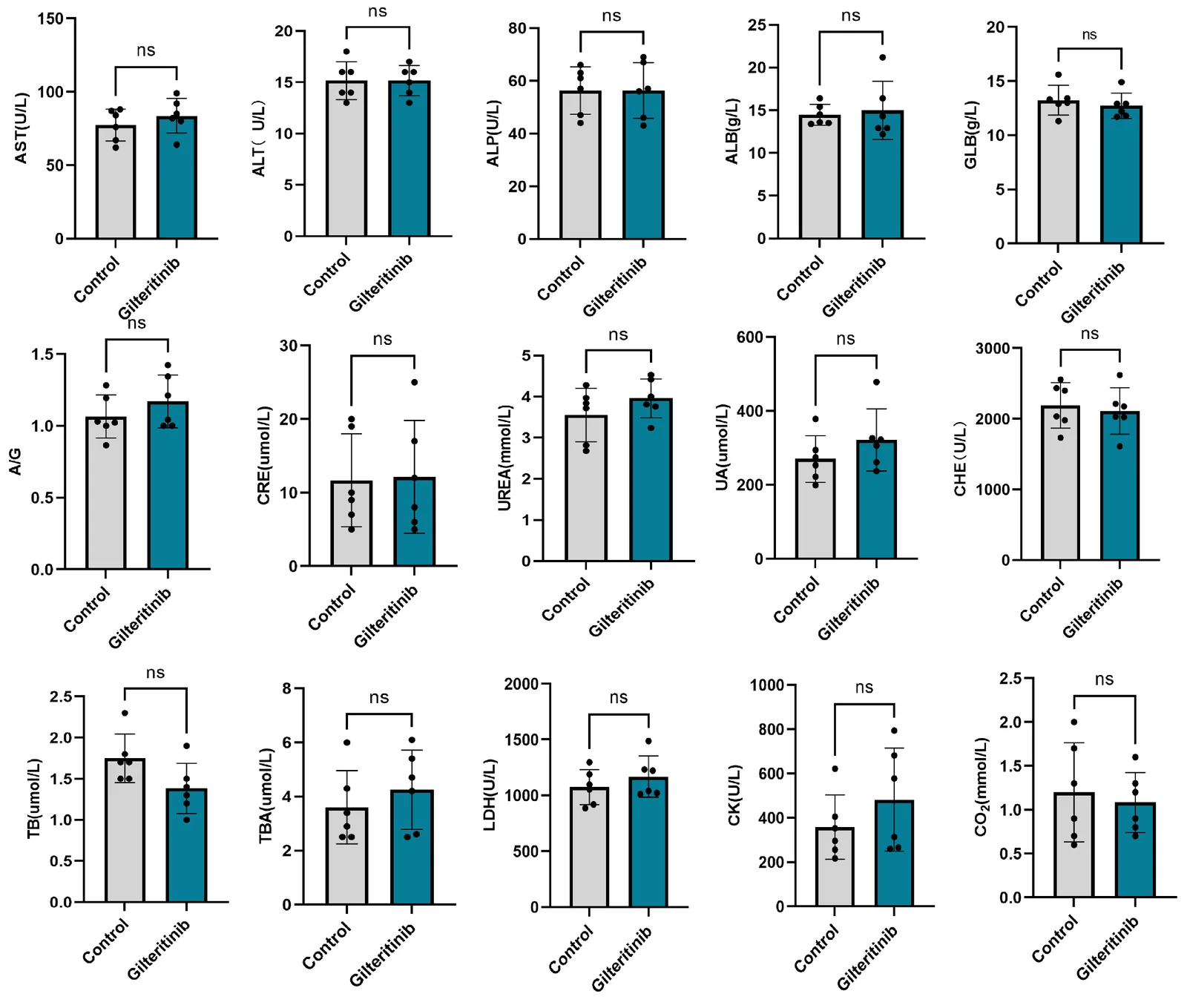
Serum biochemical evaluation in Hepa1-6 tumor-bearing mice. Serum biochemical parameters were assessed in mice bearing Hepa1-6 cell–derived tumors treated with vehicle control or gilteritinib. Liver function-related markers (AST, ALT, ALP, ALB, A/G, TB, TBA), renal function-related markers (CRE, UREA, UA), and additional indices (CHE, CK, CO_2_, GLB, LDH) are shown. n = 6 represents six individual mice per group. Data were compared using unpaired two-tailed *t*-test. ns: not significant.

## Data Availability

Data will be made available on request.

## Abbreviations

The following abbreviations are used in this manuscript:

HCC	Hepatocellular carcinoma
TKI	Tyrosine kinase inhibitor
AML	Acute myeloid leukemia
FLT3	FMS-like tyrosine kinase 3
STAT3	Signal transducer and activator of transcription 3
AKT	Protein kinase B
MAPK/ERK	Mitogen-activated protein kinase/extracellular signal-regulated kinase
PI3K	Phosphoinositide 3-kinase
mTOR	Mammalian target of rapamycin
JAK	Janus kinase
SPR	Surface plasmon resonance
EMT	Epithelial–mesenchymal transition
CCK-8	Cell Counting Kit-8
FBS	Fetal bovine serum
DMEM	Dulbecco’s Modified Eagle Medium
DMSO	Dimethyl sulfoxide
PI	Propidium iodide
FITC	Fluorescein isothiocyanate
SDS-PAGE	Sodium dodecyl sulfate–polyacrylamide gel electrophoresis
PVDF	Polyvinylidene fluoride
HRP	Horseradish peroxidase
BCA	Bicinchoninic acid
GAPDH	Glyceraldehyde-3-phosphate dehydrogenase
*K*_D_	Equilibrium dissociation constant
*k*_a_	Association rate constant
*k*_d_	Dissociation rate constant
AST	Aspartate aminotransferase
ALT	Alanine aminotransferase
ALP	Alkaline phosphatase
ALB	Albumin
A/G	Albumin/globulin ratio
TB	Total bilirubin
TBA	Total bile acid
CRE	Creatinine
UA	Uric acid
CHE	Cholinesterase
CK	Creatine kinase
GLB	Globulin
LDH	Lactate dehydrogenase
STR	Short Tandem Repeat
